# 2948. Pediatric Clinical Outcomes of MRSA PCR Utilization for Anti-MRSA De-escalation

**DOI:** 10.1093/ofid/ofad500.187

**Published:** 2023-11-27

**Authors:** Salih Demirhan, Kiriam Escobar Lee, Philip J Lee, Sharlene Sy, Brenda I Anosike

**Affiliations:** Children’s Hospital at Montefiore, Albert Einstein College of Medicine, Bronx, NY; Children’s Hospital at Montefiore, Albert Einstein College of Medicine, Bronx, NY; Children's Hospital at Montefiore, Bronx, New York; Children’s Hospital at Montefiore, Albert Einstein College of Medicine, Bronx, NY; Children's Hospital at Montefiore, Bronx, New York

## Abstract

**Background:**

Methicillin resistant *Staphylococcus aureus* (MRSA) remains a major infectious disease challenge often requiring empiric therapy. Rapid identification methods, such as MRSA polymerase chain reaction (MRSA PCR) have been increasingly utilized as a stewardship tool for antimicrobial de-escalation in adults. More recently, a growing, albeit small, number of pediatric studies have demonstrated similar promise. However, there remains a paucity of data evaluating clinical outcomes of using MRSA PCR as a stewardship tool in pediatrics.

**Methods:**

Retrospective cohort study of nasal MRSA PCRs collected from hospitalized patients ≤ 21 years of age between November 1, 2021, and February 28, 2023. Indications for testing were categorized into 4 clinical syndromes defined a priori: pneumonia; skin and soft tissue infections (SSTI); head and neck infections (HNI); and bacteremia. Each MRSA PCR test was counted per single episode. Inclusion: all those who started on an anti-MRSA agent and discontinued ≤48 hours of a negative PCR. Primary endpoint: any adverse clinical outcome defined as restarting an anti-MRSA agent in 7 days (14 days for SSTI), readmission for same indication in 30 days, escalation of ventilatory support, new inotropic support and/or transfer to intensive care unit (ICU) -all in 7 days; and in-house mortality in 30 days.

**Figure:**
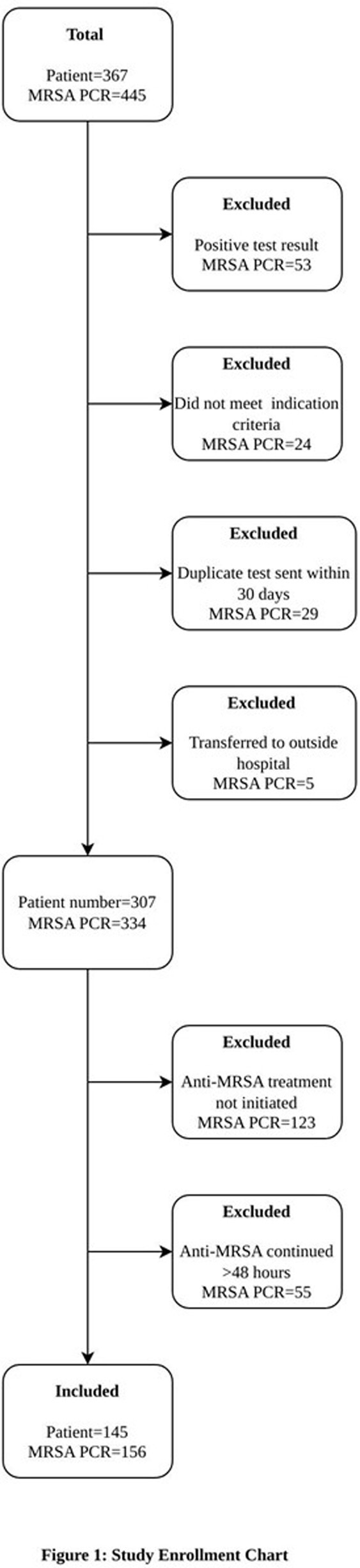

**Results:**

Total of 145 patients with 156 episodes met inclusion criteria; demographic and clinical characteristics are shown in Table 1. Pneumonia (39.1%) and SSTI (24.4%) were the most common indications for testing. 58.3%) were in the ICU at the time of testing; 48.7% received respiratory support. Vancomycin (57.0%) was the most used antibiotic. Clinical characteristics of episodes are shown in Table 2. Fifteen patients (9.6%) had any of the adverse outcomes (negative predictive value =90.4%, [95%CI: 84.6-94.5]); only 3 were restarted on an anti-MRSA agent (Table 3).

**Figure:**
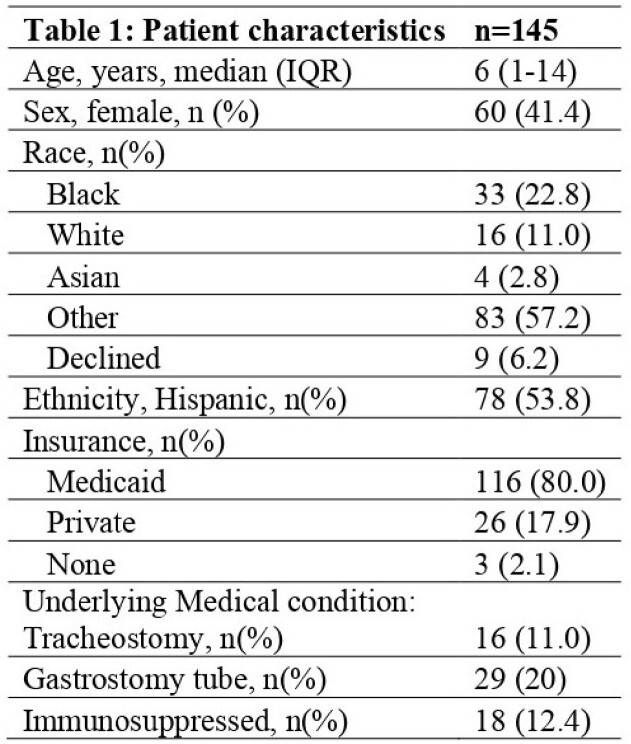

**Figure:**
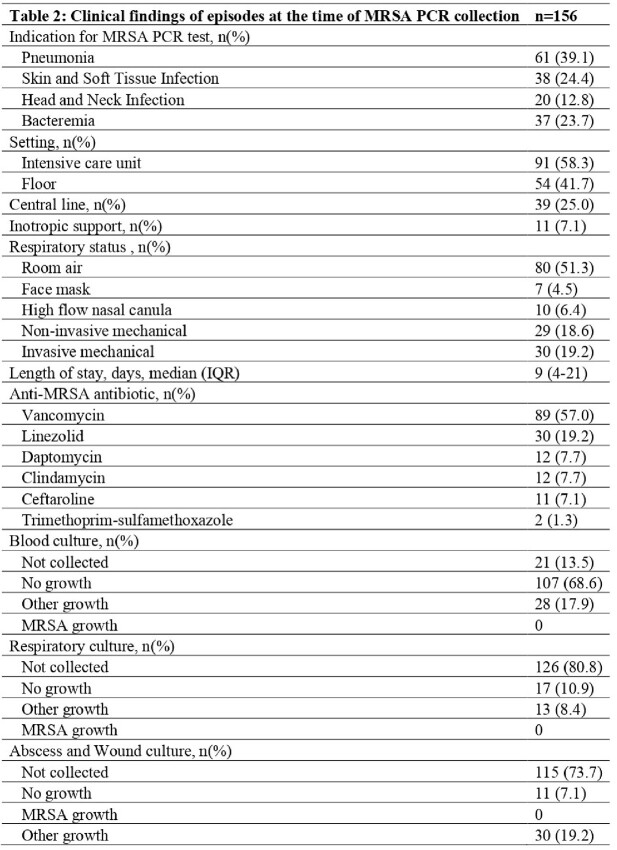

**Figure:**
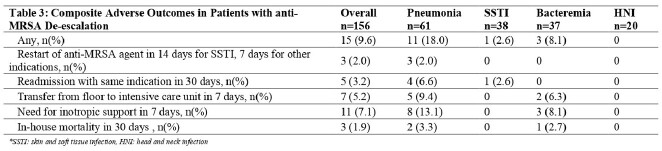

**Conclusion:**

In this single center study of pediatric patients who had a negative MRSA PCR nasal swab, none grew MRSA from cultures. Though limited, early discontinuation of anti-MRSA antibiotics was not associated with high rates of any adverse outcome suggesting the role of MRSA PCRs as a useful clinical stewardship tool in children. Larger/prospective studies are still needed.

**Disclosures:**

**All Authors**: No reported disclosures

